# The complete mitochondrial genome of *Laudakia stoliczkana stoliczkana* (Iguania; Agamidae)

**DOI:** 10.1080/23802359.2019.1644239

**Published:** 2019-07-19

**Authors:** Ya-Juan Lin, Li-Fang Peng, Shuai Li, Song Huang, Shun-Qing Lu

**Affiliations:** College of Life and Environment Sciences, Huangshan University, Huangshan, China

**Keywords:** Mitogenome, *Laudakia stoliczkana stoliczkana*, phylogenetics

## Abstract

The complete mitochondrial genome sequence of *Laudakia stoliczkana stoliczkana* was determined by shotgun sequencing. The total length of mitogenome is 16,133 bp and contains 13 protein-coding genes, 22 tRNA genes, 2 ribosome RNA genes, and 2 control regions. The base composition was 38.0% for A, 24.0% for T, 25.6% for C, and 12.4% for G. Most of the genes of *L*. *stoliczkana stoliczkana* were distributed on the H-strand, except for the ND6 subunit gene and eight tRNA genes which were encoded on the L-strand. The phylogenetic tree of *L*. *stoliczkana stoliczkana* and 16 other related species was built. The mitogenome sequence presented here will be useful to study the evolutionary relationships and genetic diversity of *Laudakia*.

*Laudakia stoliczkana*, which belongs to the genus *Laudakia* within the family Agamidae, is mainly distributed in Xinjiang province (Zhao et al. 1998; Baig et al. 2012; Wang et al. 2014). There are two subspecies included in *L. stoliczkana – L. stoliczkana stoliczkana* and *L. stoliczkana altaica*. This species is a mountain dwelling species (Baig et al. 2012). In this research, we determined and described the mitogenome of *L. stoliczkana stoliczkana* in order to obtain basic mitochondrial genetic information of this species.

The specimen of *L*. *stoliczkana stoliczkana* was collected from Meiyao village (E89.395338, N43.177625; 906 m), Turpan, Xinjiang Uygur Autonomous Region, China on 7 September 2018. It was preserved and deposited in the Museum of Huangshan University (Voucher numbers: HSR18013). The total length of the complete mitogenome (Genbank accession number: MK585009) of *L*. *stoliczkana stoliczkana* was sequenced to be 16,133 bp which consisted of 13 typical vertebrate protein-coding genes (PCGs), 22 transfer RNA (tRNA) genes, 2 ribosomal RNA (rRNA) genes, and 2 control regions (D-loop). The positions of RNA genes were predicted by the MITOS (Bernt et al. 2013) and the locations of protein-coding genes were identified by comparing with the homologous genes of other related species. The base composition was 38.0% for A, 24.0% for T, 25.6% for C, and 12.4% for G. Most of the *L*. *stoliczkana stoliczkana* mitochondrial genes are encoded on the H-strand except for the ND6 gene and 8 tRNA genes, which are encoded on the L-strand. Among the mitochondrial protein-coding genes, the ATP8 was the shortest, while the ND5 was the longest. The gene order, contents, and base composition are identical to those found in typical vertebrates (Boore 1999; Sorenson et al. 1999).

To further validate the newly determined sequences, whole mitochondrial genome sequences of *L*. *stoliczkana stoliczkana* in this study and together with other 16 related species from GenBank was used to perform the phylogenetic analysis. We aligned these sequences using Clustal X (Thompson et al. 1997). Maximum likelihood (ML) methods were used to reconstruct the phylogenetic tree (Figure 1) in http://www.phylo.org/portal2/login!input.action. The phylogenetic analysis (Figure 1) result was consistent with the previous research. It is shown that the mitogenome of this species was genetically belonging to that of *Laudakia* with a high support. It indicated that our newly determined mitogenome sequences could meet the demands and explain some evolution issues.

**Figure 1.**
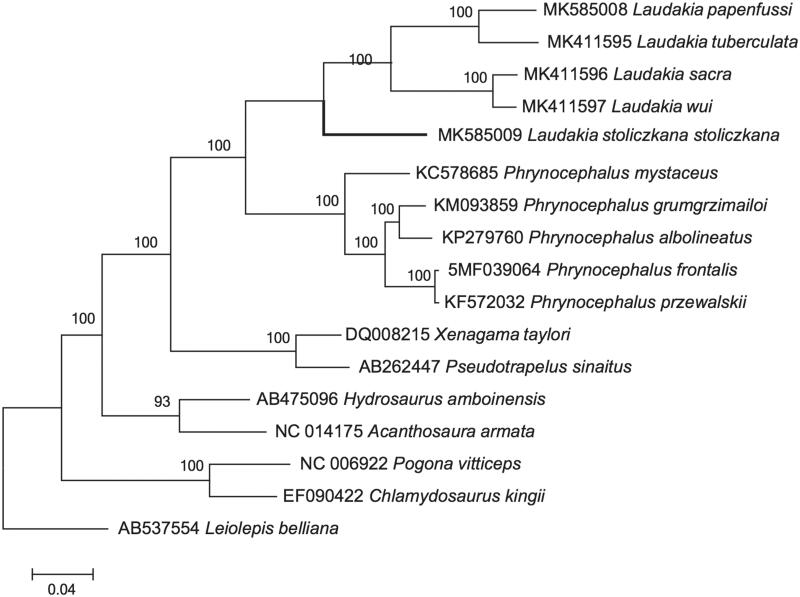
A maximum likelihood (ML) tree of *L. stoliczkana stoliczkana* in this study and other related species was constructed based on the dataset of the whole mitochondrial genome by online tool RAxML. The numbers above the branch meant bootstrap value. Bold black branches highlighted the study species and corresponding phylogenetic classification.
